# Sleep Disturbance in Adjustment Disorder and Depressive Episode

**DOI:** 10.3390/ijerph16061083

**Published:** 2019-03-26

**Authors:** Anne M. Doherty, Louisa Lorenz, Faraz Jabbar, Eamonn O’Leary, Patricia Casey

**Affiliations:** 1 Department of Adult Psychiatry, UCD School of Medicine and Medical Science, University College Dublin, Mater Misericordiae University Hospital, 62/63 Eccles Street, 7 Dublin, Ireland; l.lorenz@psychologie.uzh.ch (L.L.); Faraz.jabbar@ubc.ca (F.J.); profcasey@esatclear.ie (P.C.); 2 Department of Psychiatry, University Hospital Galway, H91 YR71 Galway, Ireland; 3 Department of Psychiatry, National University of Ireland, Galway, Newcastle, H91 TK33 Galway, Ireland; 4 Psychologisches Institut, University of Zurich, Rämistrasse 71, CH-8006 Zürich, Switzerland; 5 Department of Psychiatry, University Hospital of Northern British Columbia, 1444 Edmonton Street, Prince George, BC V2M6W5, Canada; 6 National Cancer Registry Ireland, Building 6800, Cork Airport Business Park, Kinsale Road, Cork T12 CDF7, 021 Cork, Ireland; e.oleary@ncri.ie

**Keywords:** adjustment disorder, depressive episode, sleep initiation and maintenance disorders, liaison psychiatry, diagnosis, sleep

## Abstract

*Background:* In this paper, we aimed to examine the patterns of sleep disturbance in adjustment disorder (AD) and depressive episode (DE), to examine the variables associated with sleep disturbance in AD and DE and associated impairment in functioning. *Methods:* This is a multi-centre case-control study of 370 patients: 185 patients with AD and 185 patients with a diagnosis of DE, recruited from the liaison psychiatry services of three Dublin hospitals. We examined the participants’ sleep pathology using the sleep disturbance items on the Schedule for Clinical Assessment in Neuropsychiatry, and the Inventory of Depressive Symptoms—Clinician-rated-30. *Results:* Patients with a diagnosis of AD were less likely to report disturbed sleep than those with a diagnosis of DE (*p* = 0.002). On multivariate analysis, sleep disturbance was significantly associated with greater severity of certain depressive symptoms: decreased appetite (*p* < 0.001) and psychomotor agitation (*p* = 0.009). Decreased appetite, younger age and single marital status were significantly associated with sleep disturbance in male patients, and decreased appetite and psychomotor agitation were significantly associated with sleep disturbance in female participants. *Conclusions:* This is the largest study to date which has examined sleep disturbance in adjustment disorder. Disturbance of sleep is a significant symptom in AD and may represent a potential target for treatment. With further research, patterns of sleep disturbance may be useful in differentiating AD from DE.

## 1. Introduction

Sleep disturbance is a common symptom of many psychiatric disorders and is included as a diagnostic criterion in many conditions, most notably depressive episode (DE) [1]. Adjustment disorder (AD) is defined by the World Health Organisation (WHO) in the tenth edition of The International Classification of Diseases: Classification of Mental and Behavioural Disorders (ICD-10) as a state of “subjective distress and emotional disturbance, usually interfering with social functioning and performance, and arising in the period of adaptation to a significant life change or to the consequences of a stressful life event” [1]. A diagnosis of AD requires the presence of a precipitating stressor, resolution of symptoms within six months of the termination of the stressor and the absence of another mental disorder. The ICD-10 diagnostic criteria do not specify the symptoms we expect to see in AD beyond “those found in any of the affective disorders”, but some indication of this is given in the sub-groupings of AD in ICD-10, where there are subcategories including “brief depressive reaction”, “prolonged depressive reaction”, “mixed anxiety and depressive reaction” indicate the common presentations of the condition [1]. Similarly, DSM-5 has categories “with depressed mood”, “with anxiety” and “with mixed anxiety and depressed mood” [2]. Given that AD has symptomatic overlap with depression and anxiety, it may be associated with sleep disturbance, although there is little in the literature regarding this.

AD is a common disorder in liaison psychiatry [3]. It is a disorder which has attracted some controversy regarding its role in the classification systems, and its nomenclature has undergone some transformation over the past 60 years, although it has retained its key clinical characteristics. These characteristics include symptoms common to those seen in both anxiety disorder and depressive episode. Unlike those conditions where a list of required symptoms is provided, the classification systems ICD-10 and DSM-5 do not provide a list of potential symptoms. It is well recognized that many of the biological symptoms of depression are commonly seen in AD [4,5], and certain symptoms such as suicidal ideation are equally common in both conditions [6]. Sleep is one of these symptoms, and to date there has been little research specifically examining the role of sleep in AD, whether as a symptom or indeed as a precipitant of other symptoms such as suicidal ideation.

Previous research has suggested that there may be significant differences in risk variables and in socio-demographic profile between those with a diagnosis of DE and those with a diagnosis of AD [7,8]. These have not identified sleep as a symptom associated with the diagnosis of AD, although it appears that sleep was not considered in these studies. Chellappa found an association between insomnia and increased suicidality in depressed patients [9]. Cheung, in a population of Taiwanese adolescents, found that sleep was associated with both major depression and AD but not directly associated with suicidal ideation [10].

Zimmerman found that patients with a diagnosis of AD reported more insomnia compared with those with a diagnosis of DE [11]. Mezzich found that depressive symptoms (including sleep disturbance) were more common in DE than AD, but more common in AD than in individuals with no diagnosis [12]. Lijun et al. likewise found that DE had a stronger association with sleep disturbance than AD, but despite this, those with AD had more significant early wakening [13].

Several studies have reported an association between certain life events including workplace difficulties and racial discrimination with sleep disturbance, but these studies were symptom-based and did not use a formal diagnosis of AD, or indeed any diagnosis of any mental disorder [14,15,16]. Pillai found an association with poor adaptation to stress and insomnia, but again, no formal diagnosis was used [17]. Lallukka found a significant relationship between sleep disturbance requiring hypnotic medications and stress related to family or work conflicts in women [18].

In this study we aimed to examine the patterns of sleep disturbance in AD in comparison to DE to study the variables associated with sleep disturbance in each diagnosis and to examine the relationship between sleep disturbance and disturbance in functioning.

Our hypothesis is that patients with AD are as likely to suffer from sleep disturbance as those with a diagnosis of a DE, and that there is no difference in the patterns of sleep disturbance between the two conditions.

## 2. Materials and Methods

The methods of this study have been previously described in detail and are summarized briefly here [19]. Participants were recruited from patients referred to the liaison psychiatry services at three Dublin hospitals and diagnosed by the liaison psychiatrists with either a DE or AD. Patients were excluded if they had a substance abuse disorder, cognitive impairment, psychotic symptoms, were under 18 or unable to give informed consent. Participants were interviewed at time of diagnosis, and again after six months, for stability of diagnosis.

In examining sleep, the sleep disturbance items on the Schedule for Clinical Assessment in Neuropsychiatry (SCAN) version 2, and the Inventory of Depressive Symptoms—Clinician-rated-30 (IDS-C30) were used [20,21]. SCAN is a semi-structured interview schedule created by the WHO and the gold standard in the diagnosis and measurement of mental illness [20]. Specifically, we used question 8.009 from SCAN, which examined sleep disturbance globally asking: “Have you had any trouble with sleep in the past 2 weeks?”. This is endorsed if the participant reported disturbed sleep in the preceding two weeks. For the variable of decreased function, we used ‘interference with activities due to sleep problems’ in SCAN (variable 8.021: “How much interference has there been with your everyday activities because of your problems sleeping?”). This is endorsed where the patient reported that sleep disturbance is causing the functional problems.

Both of these variables are rated on a scale from 0 to 3, scoring 0 if there is a “positive rating of absence”, scoring 1 where a symptom is present but to “such a minor degree that it is not appropriate for use in classification”, scoring 2 where the symptom “is present to a level sufficient to use in classification”, and a score of 3 is given where “the symptom is present in severe form for most of the period”. For logistic regression we divided the participants into those without the symptom (scoring 0 or 1) and compared them with those participants who endorsed the symptoms (scoring 2 or 3).

IDS-C30 was used to measure overall depressive symptoms [21]. It is a clinician administered 30 item scale, which is used to examine specific depressive symptoms in detail, each of which is rated on a scale of 0–3. It has a high level of internal consistency with a Cronbach’s alpha of 0.892.

We used the first four questions of IDS-C30, which examine specific types of sleep disturbance: “sleep onset insomnia”, “middle insomnia”, “early morning insomnia” and “hypersomnia”, each on a scale 0–3 [21]. Sleep onset insomnia was scored 0 if the participant “never takes longer than 30 min to fall asleep”, scored 1 if the participant “takes at least 30 min to fall asleep, less than half the time”, scored 2 if the participant “takes at least 30 min to fall asleep, more than half the time”, and scored 3 if the participant “takes at least 60 min to fall asleep, more than half the time”. Mid-nocturnal insomnia was scored 0 if the participant “does not wake up at night”, scored 1 if the participant reports a “restless, light sleep with few awakenings”, scored 2 if the participant “wakes up at least once a night, but goes back to sleep easily”, and scored 3 if the participant “wakes up more than once a night and stays awake for 20 min or more, more than half the time”. Early morning insomnia was scored 0 if the participant “less than half the time, awakens no more than 30 min before necessary”, scored 1 if the participant: “more than half the time, awakens more than 30 min before need be”, scored 2 if the participant “awakens at least one hour before need be, more than half the time,”, and scored 3 if the participant “awakens at least two hours before need be, more than half the time”. Hypersomnia was scored 0 if the participant “sleeps no longer than 7–8 h/night, without naps”, scored 1 if the participant “sleeps no longer than 10 h in a 24 h period (include naps)”, scored 2 if the participant “sleeps no longer than 12 h in a 24 h period (include naps)” and scored 3 if the participant “sleeps longer than 12 h in a 24 h period (include naps)”.

We explored the relationship of disturbed sleep to diagnosis, depressive symptoms and other variables related to this relationship, including life events, social functioning and personality. In multivariate analysis, for depressive symptoms we used the total score of IDS-C30, minus those scores derived from the sleep items [21].

The power calculations performed were based on methods described by Smith and Morrow [22]. To have 95% confidence of detecting a difference in depressive symptomatology of similar magnitude to that detected in Casey et al. [23], at a significance level of *p* < 0.05, we required 185 individuals with AD and 185 individuals with DE. Statistics were calculated using SPSSv24 [24]. Univariate analysis was conducted using an independent samples t-test, Mann‒Whitney U test and chi-square test. The outlier analysis using the Mahalanobis distance detected no multivariate outliers in the data. Multivariate analysis was conducted using logistic regression with the variables disturbance of sleep and impairment of function as the binary dependent variables respectively, as described above. The independent variables entered into each model were sex, age, marital status and those variables which were found to have a significant association with the dependent variable. We decided to combine the two diagnostic categories in order to examine sleep disturbance and related functional impairment in the population as a whole, as this study was underpowered for this post hoc analysis.

Ethical approval was granted by the Mater University Hospital Research Ethics Committee Ref/1/378/1146.

## 3. Results

A total of 370 patients were recruited to this study. Of these, 185 (50%) had a diagnosis of AD; the remainder were diagnosed with DE. A majority, 235 (63.5%) participants were female. The average age was 43.8 years (SD 14.2), and the mean age of patients with a diagnosis of AD were older (but not significantly older) than those diagnosed with DE. There were no significant differences between the two diagnostic groups in the socio-demographic variables of gender and marital status. Patients with a diagnosis of AD were significantly less likely to report disturbed sleep than those with a diagnosis of DE (Table 1). There were no significant differences in early onset sleep disturbance or oversleeping, but mid-nocturnal sleep disturbance and early wakening were significantly more commonly reported in patients with a diagnosis of DE, who were likewise significantly more likely to report disturbance of functioning due to sleep problems than those with AD. Patients with AD were significantly less likely (*p* < 0.001) to have been prescribed hypnotics. When we examined the patterns of disturbed sleep by gender, there were no significant differences between male and female participants in this study (Supplementary Table S1).

When participants who reported disturbed sleep were compared to those who did not, the group with disturbed sleep reported significantly more depressive symptoms and were significantly more likely to have a diagnosis of DE rather than AD (Table 2). They were significantly more likely to report other depressive symptoms as measured by the IDS-C30 including disturbance of mood, decreased appetite, decreased concentration, negative outlook towards self and future, poor energy, decreased enjoyment, reduced sexual interest, psychomotor agitation and leaden paralysis. They had higher mean scores on the IDS-C30, indicating a greater burden of depressive symptoms.

Participants who reported disturbance of function due to disturbed sleep reported significantly more depressive symptoms compared with those who did not report disturbance of functioning. They were significantly more likely to have a diagnosis of DE rather than AD. They had higher mean scores on IDS-C30, indicating a greater burden of depressive symptoms. They were significantly more likely to report other depressive symptoms as measured by the IDSC-C30 including disturbance of mood, decreased appetite, decreased concentration, negative outlook towards self and future, poor energy, decreased enjoyment, reduced sexual interest and interpersonal sensitivity (Table 3).

On multivariate analysis, where sleep disturbance was the dependent variable, it was significantly associated with the following specific depressive symptoms: decreased appetite, and psychomotor in the study population as a whole, after controlling for age, gender and marital status (Table 4). We decided to combine the two diagnostic categories in order to examine sleep disturbance and related functional impairment in the population as a whole, as when treated separately, the findings were non-significant, probably due to this study being underpowered for this post hoc analysis.

When the sample was divided by gender, there were differences between the male and female participants. Among female participants, decreased appetite and psychomotor agitation were associated with sleep disturbance. Among male participants, younger age, single marital status and decreased appetite were associated with sleep disturbance.

We performed multivariate analysis with disturbance of function due to disturbed sleep as the dependent variable. In the population as a whole, it was significantly associated with a diagnosis of DE and single marital status, and with the following specific depressive symptoms: decreased appetite, decreased energy and psychomotor agitation, after controlling for age and gender (Table 5). When the sample was divided by gender, there were differences between the male and female participants. Among female participants, a clinical diagnosis of DE, low mood, decreased appetite and reduced enjoyment were associated with greater impairment of function attributed to sleep disturbance. Among male participants, younger age, decreased appetite, psychomotor agitation and reduced leaden paralysis were associated with sleep disturbance.

## 4. Discussion

In examining the patterns of sleep disturbance in AD and DE, we found that certain symptoms were equally common in both conditions, namely “sleep onset insomnia” and “hypersomnia”, while others (middle insomnia and early morning insomnia) were more common in DE. Depressive symptoms were significantly associated with sleep disturbance in the two diagnostic groups, both combined and separately. On multivariable analysis, decreased appetite and psychomotor agitation were associated with sleep disturbance. A diagnosis of DE, decreased appetite, decreased energy and psychomotor agitation were associated with impairment of function due to disturbed sleep.

We found that sleep disturbance was associated with different variables in male and female individuals with adjustment disorder or depression in a liaison psychiatry setting. Decreased appetite was significantly associated with sleep disturbance in both genders. As both disturbance of sleep and disturbance of appetite are important biological symptoms of depression, this is not unexpected [1,2]. In addition, younger age and single marital status were associated with sleep disturbance in male patients, and psychomotor agitation was significantly associated with sleep disturbance in female participants. These findings have not previously been examined in the literature and our findings suggest that for females with AD and DE, symptoms of depression are most associated with sleep disturbance, whereas psychosocial variables have a greater impact in males with AD and DE.

Decreased appetite was likewise significantly associated with impairment of functioning attributable to sleep disturbance in both genders. In addition, a clinical diagnosis of DE, low mood, and reduced enjoyment were significantly associated with impairment of functioning attributable to sleep disturbance in female participants, and younger age, psychomotor agitation and reduced leaden paralysis were associated with impairment of functioning attributable to sleep disturbance in male patients. These findings are consistent with existing evidence which has demonstrated gender-based differences in the covariates of both sleep disturbance and associated functional impairment [25,26]. Like Chasens et al., we found significant gender-based expression of impaired sleep on functional outcomes [25]. Previous work by Hyde has suggested that any gender-based differences in functional impairment due to sleep disturbance were likely to be small [27]. In contrast to Hyde, it is difficult to comment on the strength of these differences given that the study was not powered for these post hoc analyses. 

With respect to our hypothesis, that patients with AD are as likely to suffer from sleep disturbance as those with a diagnosis of DE, we found that sleep disturbance is common in both conditions but significantly more common in DE. We found that a diagnosis of DE was associated with sleep disturbance in females, but that there was no difference between the two diagnoses in males.

This study confirms that sleep disturbance is a common symptom in AD and one which is associated with significant disturbance of functioning. It also indicates gender-related differences in this cohort of patients with low mood attending a liaison psychiatry service. No previous study has examined the patterns of sleep disturbance in this level of detail: indeed, only two have examined sleep disturbance in AD at all, and these studies have produced conflicting evidence. Like Zimmerman, we found significant degrees of reported sleep disturbance in AD, and like Mezzich, we found greater symptom severity in DE compared with AD [11,12]. Certain symptoms were similarly common in both conditions, such as sleep onset insomnia and hypersomnia. Others, insomnia and early morning insomnia, were more common in DE. The association of functional impairment attributable to disturbed sleep with specific depressive symptom severity in AD and DE on multivariable testing has not been previously demonstrated.

This study is one of few which have examined the association of AD with sleep disturbance, and the first to examine the sleep pattern seen in AD and to investigate the other variables associated with both sleep disturbance and functional impairment associated with sleep disturbance. It is one of the first to examine gender related differences in sleep in a cohort of patients with AD. This study included a large number of patients with AD: the relatively large sample size derived from our power calculations. This is the largest study yet to have been conducted examining sleep disturbance in patients with AD and DE. Previous studies examining the relationship between AD and sleep have been of smaller sample size [28]. This study used instruments which have been validated in similar populations to assess a broad range of parameters including depressive symptoms [21], which allowed us to control for a wide range of confounding variables. A systematic review found that sleep disturbance is associated with increased suicidal behaviour, and although this was not a finding of our study, it is an important target for treatment in people with impairment of psychosocial function in adjustment disorder [29]. No previous studies have examined gender-based differences in sleep disturbance in AD.

The limitations of this study include the use of clinical diagnosis. We chose the clinical diagnosis from the two options available (clinical diagnosis and SCAN diagnosis) as the gold standard for the purpose of this study. Clinical diagnoses are informed by the ICD-10 diagnostic guidelines in a broad fashion and take into account the context within which symptoms arise. This optimizes the applicability of findings to everyday clinical practice. Unlike clinical diagnosis, SCAN diagnosis looks at symptoms only, without taking account of context, and context is essential in making a diagnosis of AD. We expected that as a result of this inherent flaw in the diagnostic instruments, the use of SCAN would result in a conflation of AD with DE, and as a result would not be useful in distinguishing between the two diagnoses [5]. In our comparison of the two diagnoses this proved to be well-founded, as 73.4% of those participants with a clinical diagnosis of AD were diagnosed with DE using SCAN, resulting in a sensitivity of 91.8% and a specificity of 57.2%. The heterogeneity of clinical presentation in AD, including fluctuating psychological symptoms, behavioural disturbances and physical symptoms which can characterize certain clinical presentations with AD, adds to the complexity of this diagnosis [28]. Other limitations include the small numbers with sleep disturbance.

Finally, participants were recruited from a consultation-liaison psychiatry population in two general hospitals and one maternity hospital. While this might be regarded as limiting the generalizability of findings to this population and being of less relevance to other populations such as community-based mental health services or primary care, it has the merit of focusing on a population in which AD is particularly common and thus is well appointed to provide useful information for consultation-liaison psychiatry teams [29]. This diversity of participants also spans the full range of severity, from less severe cases treated as outpatients to more severe cases presenting to the emergency department with acute and severe symptoms.

This paper is novel in its examination of the disturbance of sleep which occurs not only in DE, but also in AD. We have demonstrated an association between sleep disturbance and other depressive symptoms in both diagnostic groups and that where sleep disturbance is present, patients report a greater severity of depressive symptoms, irrespective of diagnosis. Sleep disturbance may represent a new focus for treatments such as cognitive behavioural therapy, which has a strong evidence base in the treatment of sleep disturbance.

## 5. Conclusions

This study found that while sleep disturbance is more severe in DE, it is also common in AD and is frequently associated with disturbance of function. It is a significant symptom in AD and one which may be amenable to a behavioural approach, given that psychotropics have a poor evidence base within this diagnostic group. Further research is required to identify if patterns of sleep disturbance may be useful in the clinical challenge of differentiating AD from DE.

## Figures and Tables

**Table 1:** Demographic and clinical characteristics of patients divided by diagnosis.

Characteristics		Total	Adjustment Disorder	Depressive Episode	*p* Value
**Age**	Mean (SD)	43.8 (14.2)	43.5 (14.5)	44.1 (13.9)	0.676 ^a^
Gender	Male (%)	135 (36.5)	66 (48.9)	69 (51.1)	0.746 ^b^
	Female (%)	235 (63.5)	119 (50.6)	116 (49.4)	
Marital Status	Single, *n* (%)	131 (36.0)	65 (35.5)	66 (36.4)	0.422 ^b^
	Married/Cohabiting, *n* (%)	163 (44.8)	78 (42.6)	85 (47.9)	
	Sep/Div/Widowed, *n* (%)	70 (19.2)	40 (21.9)	30 (16.6)	
**Depressive symptoms: mean IDS-C30 total score, range 0–90**	**Mean (SD)**	**34.9 (12.7)**	**30.8 (12.3)**	**38.8 (11.9)**	**<0.001 ^a^**
**Depressive symptoms: mean IDS-C30 total score minus sleep item, range 0–78** **%**	**Mean (SD)**	**30.1 (11.0)**	**26.6 (10.6)**	**33.5 (10.4)**	**<0.001 ^a^**
Sleep disturbance, range 0–3 *	**Mean (SD)**	**1.5 (1.0)**	**1.4 (1)**	**1.7 (0.9)**	**0.002 ^a^**
Impairment of function, range 0–3 *	**Mean (SD)**	**1.1 (0.9)**	**0.9 (0.8)**	**1.3 (0.9)**	**<0.001 ^a^**
Early insomnia, range 0–3%	Mean (SD)	1.6 (1.1)	1.5 (1.1)	1.7 (1)	0.251 ^a^
**Mid nocturnal insomnia, range 0–3** **%**	**Mean (SD)**	**1.6 (1.1)**	**1.4 (1.1)**	**1.7 (1)**	**0.003 ^a^**
**Early wakening, range 0–3** **%**	**Mean (SD)**	**1.4 (1.2)**	**1.1 (1.1)**	**1.6 (1.1)**	**<0.001 ^a^**
Hypersomnia, range 0–3%	Mean (SD)	0.3 (0.6)	0.2 (0.6)	0.3 (0.7)	0.072 ^a^
**Hypnotic use**	**N (%)**	**135 (38.8)**	**46 (26.6)**	**89 (50.9)**	**<0.001 ^b^**

^a^ = Independent samples *t*-test; ^b^ = chi square; % variables from Inventory of Depressive Symptoms—Clinician-rated-30 (IDS-C30), higher scores denote greater symptoms burden; * variables from the Schedule for Clinical Assessment in Neuropsychiatry (SCAN), higher scores denote greater symptoms burden.

**Table 2:** Demographic and clinical characteristics of patients divided by sleep disturbance.

Characteristics		Total *n* = 344	Sleep Disturbance * *n* = 181	No Sleep Disturbance * *n* = 163	*p* Value
Age	Mean (SD)	43.6 (14.3)	43.5 (13.9)	43.7 (14.9)	0.865 ^a^
Gender	Male (%)	120 (34.9)	70 (38.7)	50 (30.7)	0.120 ^b^
	Female (%)	224 (65.1)	111 (61.3)	113 (69.3)	
Marital Status	Single, *n* (%)	119 (35.2)	64 (36.2)	55 (34.2)	0.781 ^b^
	Married/Cohabiting, *n* (%)	155 (45.9)	78 (44.1)	77 (47.8)	
	Sep/Div/Widowed, *n* (%)	64 (18.9)	35 (19.8)	29 (18.0)	
**Depressive symptoms: mean IDS-C30 total score, range 0–90** **%**	**Mean (SD)**	**34.9 (12.7)**	**40.2 (11.4)**	**29 (11.4)**	**<0.001 ^a^**
**Depressive symptoms: mean IDSC-30 total minus sleep items, range 0–78** **%**	**Mean (SD)**	**30.1 (11)**	**34.2 (10.3)**	**25.6 (10.1)**	**<0.001 ^a^**
**Clinical diagnosis**	**Adjustment Disorder (%)**	**172 (50)**	**77 (42.5)**	**95 (58.3)**	**0.004 ^b^**
	**Depressive episode (%)**	**172 (50)**	**104 (57.5)**	**68 (41.7)**	
**Other depressive symptoms from IDS-C30** **%** **Mood sad**	**Mean (SD)**	**1.9 (0.9)**	**2.0 (0.8)**	**1.7 (0.9)**	**0.002 ^a^**
**Mood irritable**	**Mean (SD)**	**1.5 (0.9)**	**1.6 (0.8)**	**1.2 (0.9)**	**<0.001 ^a^**
**Mood anxious**	**Mean (SD)**	**1.7 (0.9)**	**1.8 (0.9)**	**1.4 (0.9)**	**<0.001 ^a^**
**Reactivity of mood**	**Mean (SD)**	**1.1 (0.9)**	**1.2 (0.9)**	**0.9 (0.9)**	**0.003 ^a^**
Mood variation	Mean (SD)	**0.9 (1.0)**	1.0 (1.0)	0.8 (1.0)	0.112 ^a^
**Mood worse in morn**	**Mean (SD)**	**0.5 (0.8)**	**0.4 (0.7)**	**0.7 (0.9)**	**0.003 ^a^**
Mood variation due to environment	Mean (SD)	0.3 (0.5)	0.3 (0.5)	0.3 (0.5)	0.142 ^a^
Quality of mood	Mean (SD)	1.4 (1.0)	1.4 (0.9)	1.2 (1.1)	0.057 ^a^
**Appetite decreased**	**Mean (SD)**	**1.0 (0.9)**	**1.2 (0.9)**	**0.7 (0.9)**	**<0.001 ^a^**
Appetite increased	Mean (SD)	0.3 (0.9)	0.3 (0.9)	0.4 (1.0)	0.211 ^a^
Weight decrease 2 weeks	Mean (SD)	0.7 (0.9)	0.7 (0.9)	0.6 (1.0)	0.431 ^a^
Weight increase 2 weeks	Mean (SD)	0.3 (0.7)	0.3 (0.6)	0.4 (0.9)	0.105 ^a^
**Concentration/decision making**	**Mean (SD)**	**1.5 (0.8)**	**1.6 (0.8)**	**1.3 (0.8)**	**0.001 ^a^**
**Outlook self**	**Mean (SD)**	**1.3 (1.0)**	**1.4 (1.0)**	**1.1 (0.9)**	**0.016 ^a^**
**Outlook future**	**Mean (SD)**	**1.6 (0.9)**	**1.7 (0.9)**	**1.5 (0.9)**	**0.025 ^a^**
**Involvement**	**Mean (SD)**	**1.6 (0.9)**	**1.7 (0.9)**	**1.4 (1.0)**	**0.008 ^a^**
**Energy/fatigeability**	**Mean (SD)**	**1.7 (1.0)**	**1.6 (0.8)**	**1.3 (0.9)**	**0.028 ^a^**
**Pleasure/enjoyment**	**Mean (SD)**	**1.5 (0.9)**	**1.6 (0.8)**	**1.3 (0.9)**	**0.009 ^a^**
**Sexual interest**	**Mean (SD)**	**1.7 (1.0)**	**1.8 (0.9)**	**1.4 (1.0)**	**<0.001 ^a^**
Psychomotor slowing	Mean (SD)	0.6 (0.7)	0.6 (0.8)	0.6 (0.7)	0.895 ^a^
**Psychomotor agitation**	**Mean (SD)**	**0.8 (0.8)**	**0.8 (0.8)**	**0.6 (0.7)**	**0.002 ^a^**
Somatic complaints	Mean (SD)	0.9 (0.8)	0.9 (0.9)	0.9 (0.8)	0.488 ^a^
Sympathetic arousal	Mean (SD)	0.8 (0.9)	0.8 (0.8)	0.8 (1.2)	0.854 ^a^
Panic/phobic symptoms	Mean (SD)	0.9 (0.9)	1.0 (0.9)	0.8 (0.8)	0.061 ^a^
GIT	Mean (SD)	0,7 (0.9)	0.7 (1.0)	0.6 (0.7)	0.9 ^a^
Interpersonal sensitivity	Mean (SD)	1.5 (0.9)	1.5 (1.0)	1.4 (0.8)	0.074 ^a^
**Leaden paralysis**	**Mean (SD)**	**1.2 (0.9)**	**1.4 (0.9)**	**1.0 (0.8)**	**0.001 ^a^**

^a^ = Independent samples *t*-test; ^b^ = chi square; * Schedule for Clinical Assessment in Neuropsychiatry (SCAN), variable 8.009; % variables from the Inventory of Depressive Symptoms—Clinician-rated-30 (IDS-C30): higher scores denote greater symptoms burden.

**Table 3:** Demographic and clinical characteristics of patients divided by disturbance in function attributed to sleep difficulties.

Characteristics		Total *n* = 345	Functional Difficulties Due to Sleep Disturbance * *n* = 137	No Functional Problems Due to Sleep Disturbance * *n* = 208	*p* Value
Age	Mean (SD)	43.6 (14.3)	43.5 (14.5)	43.7 (14.3)	0.921 ^a^
Gender	Male *n* (%)	122 (35.4)	50 (36.5)	72 (34.6)	0.721 ^b^
	Female *n* (%)	223 (64.6)	87 (63.5)	136 (65.4)	
Marital Status	Single, *n* (%)	119 (35.1)	43 (32.1)	76 (37.1)	0.643 ^b^
	Married/Cohabiting, *n* (%)	155 (45.7)	64 (47.8)	91 (44.4)	
	Sep/Div/Widowed, *n* (%)	65 (19.2)	27 (20.1)	38 (18.5)	
**Depressive symptoms: mean IDS-C30 total score, range 0–90** **%**	**Mean (SD)**	**35 (12.6)**	**39.2 (12.4)**	**32.3 (12)**	**<0.001 ^a^**
**Depressive symptoms: mean IDS-C30 total score minus sleep item, range 0–78** **%**	**Mean (SD)**	**30.2 (11)**	**33.7 (10.7)**	**27.9 (10.5)**	**<0.001 ^a^**
**Clinical diagnosis**	**Adjustment Disorder (%)**	**175 (50.7)**	**49 (35.8)**	**121 (58.2)**	**<0.001 ^b^**
	**Depressive episode (%)**	**172 (50)**	**104 (57.5)**	**68 (41.7)**	
**Other depressive symptoms from IDS-C30** **%** **Mood sad**	**Mean (SD)**	**1.9 (0.9)**	**2.2 (0.8)**	**1.7 (0.9)**	**<0.001 ^a^**
**Mood irritable**	**Mean (SD)**	**1.5 (0.9)**	**1.8 (0.8)**	**1.3 (0.9)**	**<0.001 ^a^**
**Mood anxious**	**Mean (SD)**	**1.7 (0.9)**	**1.9 (0.9)**	**1.5 (0.9)**	**<0.001 ^a^**
**Reactivity of mood**	**Mean (SD)**	**1.1 (0.9)**	**1.3 (0.9)**	**1.0 (0.9)**	**0.014 ^a^**
**Mood variation**	**Mean (SD)**	**0.9 (1.0)**	**1.1 (1.1)**	**0.8 (1.0)**	**0.017 ^a^**
**Mood worse in morn**	**Mean (SD)**	**0.5 (0.8)**	**0.3 (0.7)**	**0.6 (0.8)**	**0.003 ^a^**
Mood variation due to environment	Mean (SD)	0.3 (0.5)	0.2 (0.4)	0.3 (0.5)	0.079 ^a^
**Quality of mood**	**Mean (SD)**	**1.4 (1.0)**	**1.5 (0.9)**	**1.3 (1.0)**	**0.011 ^a^**
**Appetite decreased**	**Mean (SD)**	**1.0 (0.9)**	**1.3 (0.9)**	**0.8 (0.9)**	**<0.001 ^a^**
Appetite increased	Mean (SD)	0.3 (0.9)	0.2 (0.9)	0.4 (0.9)	0.165 ^a^
Weight decrease 2 weeks	Mean (SD)	0.7 (0.9)	0.7 (0.9)	0.7 (1.0)	0.916 ^a^
Weight increase 2 weeks	Mean (SD)	0.3 (0.7)	0.2 (0.6)	0.4 (0.8)	0.052 ^a^
**Concentration/decision making**	**Mean (SD)**	**1.5 (0.8)**	**1.7 (0.7)**	**1.4 (0.8)**	**0.001 ^a^**
**Outlook self**	**Mean (SD)**	**1.3 (1.0)**	**1.5 (1.0)**	**1.2 (0.9)**	**0.003 ^a^**
**Outlook future**	**Mean (SD)**	**1.6 (0.9)**	**1.8 (0.9)**	**1.5 (0.9)**	**0.003 ^a^**
**Involvement**	**Mean (SD)**	**1.6 (0.9)**	**1.8 (0.9)**	**1.4 (0.9)**	**<0.001 ^a^**
**Energy/fatiguability**	**Mean (SD)**	**1.7 (1.0)**	**1.9 (0.8)**	**1.6 (0.8)**	**<0.001 ^a^**
**Pleasure/enjoyment**	**Mean (SD)**	**1.5 (0.9)**	**1.7 (0.9)**	**1.3 (0.8)**	**<0.001 ^a^**
**Sexual interest**	**Mean (SD)**	**1.7 (1.0)**	**1.9 (0.9)**	**1.6 (1.0)**	**0.004 ^a^**
Psychomotor slowing	Mean (SD)	0.6 (0.7)	0.6 (0.8)	0.6 (0.7)	0.856 ^a^
**Psychomotor agitation**	**Mean (SD)**	**0.8 (0.8)**	**1.0 (0.9)**	**0.6 (0.8)**	**<0.001 ^a^**
Somatic complaints	Mean (SD)	0.9 (0.8)	1.0 (0.9)	0.9 (0.8)	0.328 ^a^
Sympathetic arousal	Mean (SD)	0.8 (0.9)	0.8 (0.9)	0.7 (1.0)	0.352 ^a^
Panic/phobic symptoms	Mean (SD)	0.9 (0.9)	1.0 (0.9)	0.9 (0.9)	0.286 ^a^
GIT	Mean (SD)	0,7 (0.9)	0.7 (1.1)	0.6 (0.7)	0.331 ^a^
**Interpersonal sensitivity**	**Mean (SD)**	**1.5 (0.9)**	**1.6 (1.0)**	**1.4 (0.9)**	**0.038 ^a^**
Leaden paralysis	Mean (SD)	1.2 (0.9)	1.3 (1.0)	1.2 (0.9)	0.332 ^a^

^a^ = Independent samples *t*-test; ^b^ = chi square; * Schedule for Clinical Assessment in Neuropsychiatry (SCAN), variable 8.021; % variables from the Inventory of Depressive Symptoms—Clinician-rated-30 (IDS-C30): higher scores denote greater symptoms burden.

**Table 4:** Multivariate analysis: logistic regression, with sleep disturbance as the dependent binary variable, in AD and DE combined (i.e., the entire sample).

	Total			Male (*n* = 135)			Female (*n* = 235)		
	*p*	B	CI	*p*	B	CI	*p*	B	CI
Age	0.218	−0.002	−0.006–0.001	**0.013**	**−0.059**	**0.900–0.988**	0.440	−0.012	−0.959–1.018
Gender	0.767	−0.015	−0.115–0.085	-	-	-	-	-	-
Marital status	0.155	0.024	−0.012–0.077	**0.002**	**−2.956**	**0.008–0.350**	0.783	−0.117	−0.387–2.045
Clinical diagnosis	0.101	0.084	−0.016–0.184	0.851	−0.144	0.193–3.893	0.087	−0.788	−0.184–1.122
Depressed mood %	0.394	0.029	−0.044–0.101	0.197	0.637	0.719–4.975	0.655	–0.136	−0.480–1.587
**Decreased appetite %**	**<0.001**	**0.103**	**0.048–0.159**	**0.009**	**1.546**	**1.462–15.119**	**0.009**	**0.794**	**1.217–4.019**
Outlook for future %	0.979	0.001	−0.062–0.063	0.706	0.155	0.522–2.614	0.956	−0.016	−0.565–1.716
Energy %	0.492	−0.022	−0.094–0.05	0.174	0.646	0.752–4.836	0.832	−0.066	−0.506–1.729
Pleasure/enjoyment %	0.431	−0.032	−0.112–0.048	0.810	0.138	−0.371–3.551	0.149	0.472	−0.845–3.043
Sexual interest %	0.098	0.059	0.003–0.12	0.910	−0.050	0.398–2.271	0.570	0.146	0.7–1.911
**Psychomotor agitation %**	**0.009**	**0.077**	**0.017–0.137**	0.459	0.331	0.580–3.342	**0.181**	**−0.341**	**0.432–1.171**
Leaden paralysis %	0.061	0.062	0.001–0.124	0.399	−0.394	0.270–1.686	0.191	0.402	0.819–2.728

% variables from IDS-C30, higher scores denote greater symptom burden.

**Table 5:** Multivariate analysis: logistic regression, with disturbance in function due to sleep problems as the dependent binary variable, in AD and DE combined (i.e., the entire sample).

	Total			Male (*n* = 135)			Female (*n* = 235)		
	*p*	B	CI	*p*	B	CI	*p*	B	CI
Age	0.716	−0.001	−0.008–0.005	**0.412**	**−0.014**	**0.954–1.020**	0.846	0.002	−0.98–1.025
Gender	0.963	−0.004	−0.182–0.191	−	−	−	−	-	-
Marital status	**0.038**	**0.089**	**0.005–0.173**	0.293	−0.529	0.220–1.579	0.831	−0.070	−0.492–1.766
Clinical diagnosis	**<0.001**	**0.365**	**0.179–0.551**	0.284	−0.564	0.203–1.595	**0.001**	**−1.174**	**0.159–0.600**
Depressed mood %	0.095	0.115	−0.02–0.25	0.147	−0.533	0.285–1.207	**0.004**	**0.766**	**−1.283–3.608**
**Decreased appetite %**	**0.005**	**0.149**	**0.046–0.252**	**0.010**	**0.743**	**1.193–3.702**	**0.054**	**0.338**	**0.994–1.977**
Outlook for future %	0.294	0.062	−0.052–0.179	0.252	0.375	0.766–2.762	0.533	−0.130	−0.585–1.320
Energy %	**0.038**	**0.142**	**−0.008–0.276**	0.064	0.806	0.955–5.255	0.117	0.364	−0.913–2.269
Pleasure/enjoyment %	0.654	0.004	−0.145–0.154	**0.007**	**1.391**	**1.467–11.017**	**0.048**	**−0.511**	**0.362–0.995**
Sexual interest %	0.329	−0.057	−0.171–0.058	0.052	−0.764	0.215–1.007	0.307	0.201	0.831–1.800
**Psychomotor agitation %**	**0.001**	**0.19**	**0.078–0.301**	**0.002**	**1.085**	**1.479–5.92**	0.069	0.349	0.973–2.067
Leaden paralysis %	0.716	−0.001	−0.008–0.005	**0.036**	**−0.612**	**0.306–1.579**	0.836	−0.044	0.632–1.450

% variables from IDS-C30, higher scores denote greater symptom burden.

## Supplementary Materials

The following are available online at https://www.mdpi.com/1660-4601/16/6/1083/s1, Table S1: Demographic and clinical characteristics of patients divided by gender.

## References

[1] World Health Organization (1992). International Classification of Diseases.

[2] American Psychiatric Association (2013). Diagnostic and Statistical Manual of Mental Disorders.

[3] Silverstone P.H. (1996). Prevalence of psychiatric disorders in medical inpatients. J. Nerv. Ment. Dis..

[4] Baumeister H., Maercker A., Casey P. (2009). Adjustment disorder with depressed mood: A critique of its DSM-IV and ICD-10 conceptualisations and recommendations for the future. Psychopathology.

[5] Casey P. (2008). Diagnosing adjustment disorder with depressive features. Expert Rev. Neurother..

[6] Polyakova I., Knobler H.Y., Ambrumova A., Lerner V. (1998). Characteristics of suicidal attempts in major depression versus adjustment reactions. J. Affect. Disord..

[7] Casey P., Jabbar F., O’Leary E., Doherty A.M. (2015). Suicidal behaviours in adjustment disorder and depressive episode. J. Affect. Disord..

[8] Kryzhanovskaya L., Canterbury R. (2001). Suicidal Behavior in Patients with Adjustment Disorders. Crisis.

[9] Chellappa S.L., Araújo J.F. (2007). Sleep disorders and suicidal ideation in patients with depressive disorder. Psychiatry Res..

[10] Chung M.S., Chiu H.J., Sun W.J., Lin C.N., Kuo C.C., Huang W.C., Chen Y.S., Cheng H.P., Chou P. (2013). Association among depressive disorder, adjustment disorder, sleep disturbance, and suicidal ideation in Taiwanese adolescent. Asia Pac. Psychiatry.

[11] Zimmerman M., Martinez J.H., Dalrymple K., Martinez J.H., Chelminski I., Young D. (2013). Is the distinction between adjustment disorder with depressed mood and adjustment disorder with mixed anxious and depressed mood valid?. Ann. Clin. Psychiatry.

[12] Mezzich J.E., Fabrega H., Coffman G.A. (1987). Multiaxial characterization of depressive patients. J. Nerv. Ment. Dis..

[13] Cui L., Li K., Sun X., Cui Z., Jiang Q., Han Y., Gao L., Zhang Y., Li J., Liu Y. (2012). A survey of sleep quality in patients with 13 types of mental disorders. Prim. Care Companion CNS Disord..

[14] Grandner M.A., Hale L., Jackson N., Patel N.P., Gooneratne N.S., Troxel W.M. (2012). Perceived racial discrimination as an independent predictor of sleep disturbance and daytime fatigue. Behav. Sleep Med..

[15] Kostev K., Rex J., Waehlert L., Hog D., Heilmaier C. (2014). Risk of psychiatric and neurological diseases in patients with workplace mobbing experience in Germany: A retrospective database analysis. Ger. Med. Sci..

[16] Signorelli M.S., Costanzo M.C., Cinconze M., Concerto C. (2013). What kind of diagnosis in a case of mobbing: Post-traumatic stress disorder or adjustment disorder?. BMJ Case Rep..

[17] Pillai V., Roth T., Mullins H.M., Drake C.L. (2014). Moderators and Mediators of the Relationship Between Stress and Insomnia: Stressor Chronicity, Cognitive Intrusion, and Coping. Sleep.

[18] Lallukka T., Arber S., Laaksonen M., Lahelma E., Partonen T., Rahkonen O. (2013). Work-family conflicts and subsequent sleep medication among women and men: A longitudinal registry linkage study. Soc. Sci. Med..

[19] Doherty A.M., Kelly B.D., Jabbar F., Casey P. (2014). Distinguishing between adjustment disorder and depressive episode in clinical practice: The role of personality disorder. J. Affect. Disord..

[20] Wing J.K., Babor T., Brugha T., Burke J., Cooper J.E., Giel R., Jablenski A., Regier D., Sartorius N. (1990). SCAN: Schedules for Clinical Assessment in Neuropsychiatry. Arch. Gen. Psychiatry.

[21] Trivedi M.H., Rush A.J., Ibrahim H.M., Carmody T.J., Biggs M.M., Suppes T., Crismon M.L., Shores-Wilson K., Toprac M.G., Dennehy E.B. (2004). The Inventory of Depressive Symptomatology, Clinician Rating (IDS-C) and Self-Report (IDS-SR), and the Quick Inventory of Depressive Symptomatology, Clinician Rating (QIDS-C) and Self-Report (QIDS-SR) in public sector patients with mood disorders: A psychometric evaluation. Psychol. Med..

[22] Smith P.G., Morrow R.H. (1986). Field Trials of Health Interventions in Developing Countries: A Toolbox.

[23] Casey P., Maracy M., Kelly B.D., Lehtinen V., Ayuso-Mateos J.-L., Dalgard O.S., Dowrick C. (2006). Can adjustment disorder and depressive episode be distinguished? Results from ODIN. J. Affect. Disord..

[24] IBM Corp (2016). IBM SPSS Statistics for Windows, Version 24.0.

[25] Chasens E.R., Morris J.L., Strollo P.J., Sereika S.M., Burke L.E., Korytkowski M. (2016). Gender differences in the response to impaired sleep in adults with diabetes. Behav. Sleep Med..

[26] Shepertycky M.R., Banno K., Kryger M.H. (2005). Differences between men and women in the clinical presentation of patients diagnosed with obstructive sleep apnea syndrome. Sleep.

[27] Hyde J.S. (2005). The gender similarities hypothesis. Am. Psychol..

[28] Strain J.J., Diefenbacher A. (2008). The adjustment disorders: The conundrums of the diagnoses. Compr. Psychiatry.

[29] Porras-Segovia A., Pérez-Rodríguez M.M., López-Esteban P., Courtet P., Barrigón M.L., López-Castromán J., Cervilla J.A., Baca-García E. (2018). Contribution of sleep deprivation to suicidal behaviour: A systematic review. Sleep Med. Rev..

