# Supplementary material for: Sleep Disturbance in Adjustment Disorder and Depressive Episode
Source: Int J Environ Res Public Health. 2019 Mar 26;16(6):1083. doi: 10.3390/ijerph16061083 (PMC6466335; doi:10.3390/ijerph16061083)
Supplement: Supplementary file 1 [file ijerph-16-01083-s001.pdf]

Supplementary Table 1: Demographic and clinical characteristics of patients divided by gender.

Characteristics		Total	Male	Female	p value
Age	Mean (SD)	43.8 (14.2)	44.1 (14.6)	43.6 (14.0)	0.753[a]
Marital Status	Single, n (%)	131 (36.0)	56 (42.1)	75 (32.5)	0.156[b]
	Married/Cohabiting, n (%)	163 (44.8)	52 (39.1)	111 (48.1)	
	Sep/Div/Widowed, n (%)	70 (19.2)	25 (18.8)	45 (19.5)	
Clinical diagnosis	Adjustment disorder (%)	135 (36.5)	66 (18.8)	69 (51.1)	0.746[b]
	Depressive episode (%)	235 (63.5)	119 (50.6)	116 (49.4)	
Depressive symptoms: mean IDSC-30 total score, range 0–90%	Mean (SD)	34.9 (12.7)	34.0 (12.1)	35.3 (13.0)	0.37[a]
Depressive symptoms: mean IDSC-30 total score minus sleep items, range 0–78%	Mean (SD)	30.1 (11.0)	29.2 (10.6)	30.5 (11.3)	0.295[a]
Sleep disturbance, range 0–3*	Mean (SD)	1.5 (1.0)	1.6 (1)	1.5 (0.9)	0.10[a]
Impairment of function, range 0–3*	Mean (SD)	1.1 (0.9)	1.1 (1.0)	1.1 (0.9)	0.77[a]
Early insomnia, range 0–3%	Mean (SD)	1.6 (1.1)	1.6 (1.1)	1.6 (1)	0.625[a]
Mid nocturnal insomnia, range 0–3%	Mean (SD)	1.6 (1.1)	1.5 (1.1)	1.6 (1.1)	0.657[a]
Early wakening, range 0–3%	Mean (SD)	1.4 (1.2)	1.4 (1.2)	1.4 (1.2)	0.7[a]
Hypersomnia, range 0–3%	Mean (SD)	0.3 (0.6)	0.3 (0.7)	0.2 (0.6)	0.139[a]
Hypnotic use	N (%)	135 (38.8)	45 (36.9)	90 (39.8)	0.592[b]

a=Independent samples t-test; b=chi square;

%variables from IDS-C30, higher scores denote greater symptoms burden;

*variables from SCAN, higher scores denote greater symptoms burden.
